# Suspension of Columbia Veterinary College

**Published:** 1884-04

**Authors:** 


### EDITORIAL NOTES.

Prof. Johne, of Dresden (Deutsche Land Presse), reports quite a number of cases of Tuberculosis in hens, which were traced to their being fed by a person having the disease that had the habit of giving the hens meat she had chewed up for the juices, but did not swallow. She was very fond of the hens, and in summer weather they would congregate about her, and frequently be seen to pick up the sputa which she coughed up. The liver, kidneys and intestines were mostly affected.

Schlampp (Wochenschrift f. Thierheilkunde) reports an interesting case in a horse of perforation of one of the guttural pouches from some unknown causes. The sack became filled and distended with food, which on account of pressure, in connection with inflammation of surrounding parts, caused the animal to die with the symptoms of glottis œdema. A retro-pharyngeal abscess was supposed to be at the bottom of the trouble.

Some ten horses are now known to have died from acute inflammatory œdema of pharynx and larynx, in some way connected with eating or breathing ensilage. These causes will be especially reported in a paper written for the succeeding number of the Journal.

Suspension of Columbia Veterinary College.—The readers of the Journal are, no doubt, aware of the fact that Columbia College has gone into retirement, whether permanent or only temporary we are unable to say, and that its students have become matriculants in the American Veterinary College.

